# Mucosal Mesenchymal Cells: Secondary Barrier and Peripheral Educator for the Gut Immune System

**DOI:** 10.3389/fimmu.2017.01787

**Published:** 2017-12-13

**Authors:** Yosuke Kurashima, Daiki Yamamoto, Sean Nelson, Satoshi Uematsu, Peter B. Ernst, Toshinori Nakayama, Hiroshi Kiyono

**Affiliations:** ^1^Division of Mucosal Immunology, Department of Microbiology and Immunology, The Institute of Medical Science, The University of Tokyo, Tokyo, Japan; ^2^Division of Clinical Vaccinology, International Research and Development Center for Mucosal Vaccines, The Institute of Medical Science, The University of Tokyo, Tokyo, Japan; ^3^Institute for Global Prominent Research, Chiba University, Chiba, Japan; ^4^Department of Mucosal Immunology, Graduate School of Medicine, Chiba University, Chiba, Japan; ^5^Department of Innovative Medicine, Graduate School of Medicine, Chiba University, Chiba, Japan; ^6^Chiba University-UC San Diego Center for Mucosal Immunology, Allergy, and Vaccines (CU-UCSD cMAV), San Diego, CA, Unites States; ^7^Division of Innate Immune Regulation, International Research and Development Center for Mucosal Vaccines, The Institute of Medical Science, The University of Tokyo, Tokyo, Japan; ^8^Center for Veterinary Sciences and Comparative Medicine, University of California, San Diego, CA, Unites States; ^9^Division of Comparative Pathology and Medicine, Department of Pathology, University of California, San Diego, CA, Unites States; ^10^Department of Immunology, Graduate School of Medicine, Chiba University, Chiba, Japan

**Keywords:** intestinal stem cells, peripheral education, fibroblasts, mucosal healing, mesenchymal cells

## Abstract

Stromal connective tissue contains mesenchymal cells, including fibroblasts and myofibroblasts, which line the tissue structure. However, it has been identified that the function of mesenchymal cells is not just structural—they also play critical roles in the creation and regulation of intestinal homeostasis. Thus, mucosal mesenchymal cells instruct intestinal immune cell education (or peripheral immune education) and epithelial cell differentiation thereby shaping the local environment of the mucosal immune system. Malfunction of the mesenchymal cell-mediated instruction system (e.g., fibrosis) leads to pathological conditions such as intestinal stricture.

## Introduction

Occurring below the mucosal mucus and membrane layer and at the forefront of host-environmental encounters, interactions between epithelial and immune cells are indispensable for the formation of the chemical, physical, and immunological barriers of the mucosal epithelium. Such interactions lead to immunophysiological functions—secretion of mucus containing anti-microbial peptides and secretory IgA antibodies, and enhancement of tight junctions—ultimately promoting intestinal homeostasis (1). These indispensable roles of the mucosal epithelial-immune cell barrier are well known due to functional studies demonstrating that disruption of barrier-associated genes (e.g., encoding MUC2 and E-cadherin) results in intestinal inflammation (2–4). Recently, however, focus has shifted toward the role of mesenchymal cell interactions with epithelial and immune cells and their effect on the formation and maintenance of intestinal homeostasis.

Mesenchymal cells are a large heterogenous population that includes fibroblasts, myofibroblasts, interstitial cells of Cajal, pericytes, many of which are within the mucosa (5). They are negative for common molecular markers for epithelial and hematopoietic cells (e.g., E-cadherin and CD45, respectively) but are positive for a combination of vimentin, CD90 (also known as THY1), S100A4, α-smooth muscle actin, desmin, smoothelin, platelet-derived growth factor (PDGF) receptor, and c-kit (6, 7) (Table 1). Most notably, the expression of α-smooth muscle actin is used to distinguish between fibroblasts and myofibroblasts as the negative and positive cells, respectively [Table 1; (5)].

**Table 1:** Characteristics of surface molecules expressed by different mesenchymal cells.^a^

	Fibroblasts	Myofibroblasts	Pericytes	Smooth muscle	Interstitial cells of Cajal
Vimentin	+	+	+	−	+
CD90	+	+	±	−	−
S100A4	+	+	−	−	−
Alpha-smooth muscle actin	−	+	+	+	−
Desmin	−	−	+	+	−
Smoothelin	−	−	+	+	−
Platelet-derived growth factor receptor	+	+	+	+	?
c-kit	−	−	−	−	+

*The expression molecules of mesenchymal cells (e.g., fibroblasts, myofibroblasts, pericytes, smooth muscle cells, and interstitial cells of Cajal) were defined*.

*^a^The table was prepared by the data described in Ref. (6, 7)*.

Although mesenchymal cells have various origins, they provide mechanical and structural support functions that are integral to intestinal morphogenesis, organogenesis, and homeostasis (8–10). In mice lacking PDGF, a necessary mesenchymal growth factor (8), intestinal myofibroblasts (pericryptal fibroblasts) are lost in the villous crypts during intestinal formation, leading to disorganization of the intestine (8). In organogenesis of lymph nodes [e.g., in Peyer’s patches (PPs) and mesenteric lymph nodes], mesenchymal cells termed lymphoid tissue organizer aid in the accumulation of lymphocytes through stimulation by lymphoid tissue inducer cells (LTi or Group 3 innate lymphoid cells) (9, 10). Therefore, mesenchymal cells play multiple essential roles in developing and preserving gut anatomical homeostasis. In addition, interstitial cells of Cajal regulate gastrointestinal motility: loss of these through mutations of *KIT* cause abnormalities in intestinal peristalsis (5). Pericytes, or parietal cells, surround capillary vessels where they are responsible for regulating stretching and vascular permeability, and perform angiogenesis through interactions with endothelial cells, as reviewed elsewhere (5, 11). Fibroblasts and myofibroblasts, the main topic of this review, are essential for the formation of the higher-order structure of tissue (e.g., gastrointestinal tract) through production of extracellular matrix (ECM) (12), and therefore play an indispensable role in tissue regeneration and restoration (12).

In recent years, it has become apparent that mesenchymal cells act on various immunocompetent cells, such as dendritic cells and mast cells, to modulate differentiation, proliferation, and the function of these cells in peripheral tissues in a process we term “peripheral education” (13–15). Furthermore, mesenchymal cells regulate epithelial lineage development in intestinal infection (16). In colonic mucosa, the CD90-positive mesenchymal cell population expressing toll-like receptors and Nod-like receptors possesses phagocytic and antigen-presenting capabilities (17). Although their antigen-presenting capabilities are not as great as those of professional antigen-presenting cells, it is suggested that mesenchymal cells are involved in the direct induction or enhancement of mucosal acquired immune responses (17). Here, we provide an overview of recent advances concerning the role of mesenchymal cells in peripheral education and epithelial membrane repair for the creation of a healthy gut immune environment.

## Mesenchymal Regulatory System for Mucosal Frontline

### Function of Mucosal Mesenchymal System in Epithelial Differentiation

Along the gut epithelial layer, which forms the first line of mucosal barrier by producing mucus containing antibacterial substances (1), microfold cells (M cells) are a gateway for the outside environment and are responsible for antigen uptake (or sampling) from the mucosal lumen (18). M cells are primarily located in the follicle-associated epithelium of PPs, a major organized lymphoid structure for the induction and regulation of the appropriate antigen-specific mucosal immune responses that confer protection and commensalism against pathogenic and beneficial antigens, respectively (9, 18). *In vivo* studies and *in vitro* organoid studies have shown that the cytokine RANKL (also known as TNFSF11) is essential for the induction of differentiation and maintenance of M cells located in the follicle-associated epithelium of PPs (19, 20). Mesenchymal cells located just below the follicle-associated epithelium are the main source of RANKL (19). A most recent study has shown that the unique type 6 collagen expressing mesenchymal cell populations producing RANKL are involved in the development of M cells (21). M cells are an entry site of antigens and luminal bacteria and antigen presentations were subsequently occurred for generating IgA in the PPs; therefore, RANKL induced M cell differentiation is imperative to the maintenance of host-microbe symbiosis (21). This type of mesenchymal instruction system for the development of mucosal immune system *via* the M cell induction is one of examples for the essential role of mesenchymal cell family for mucosal frontline upkeeping system (19, 20).

**Figure 1:**
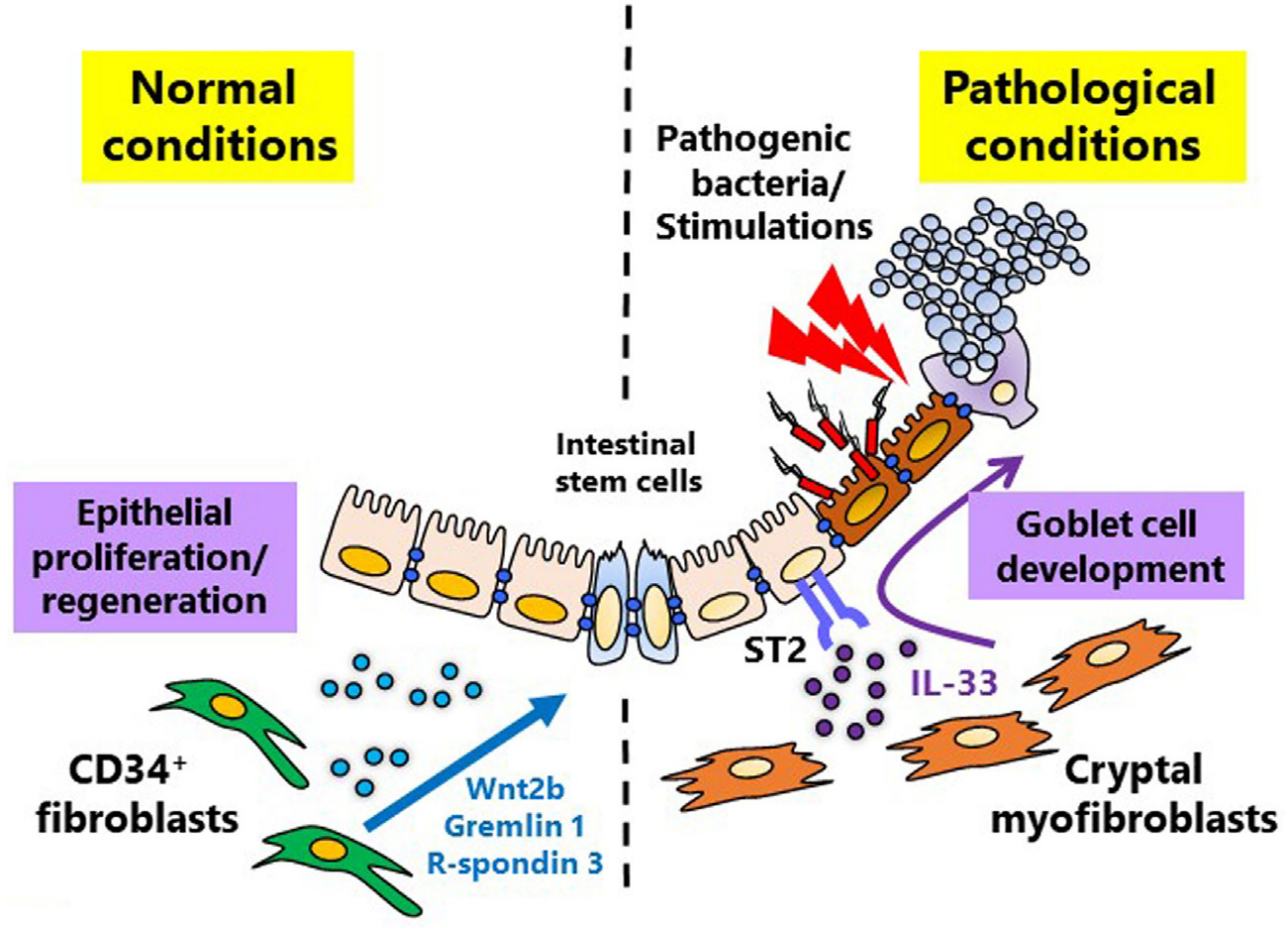
Mesenchymal cell-instructed intestinal homeostatic and pathological conditions. Under normal conditions, mesenchymal cells promote mucosal homeostasis by maintaining physiological differentiation of absorptive epithelial cells from intestinal stem cells through the production of intestinal stem cell niche factors, including Wnt2b, Gremlin 1, and R-spondin 3. During pathological conditions, including inflammation and infection, mesenchymal cells can promote the essential switch from absorptive to secretory epithelial differentiation which is mediated by interleukin-33.

In the villi, mesenchymal cells guide epithelial cell (EC) lineage differentiation in both physiological and pathological conditions (6, 22). Under the homeostatic condition, epithelial stem cells primarily differentiate into absorptive ECs, which perform the primary physiological function of the gastrointestinal tract (1), however, upon infection, epithelial stem cells shift toward secretory EC differentiation (23). In the case of bacterial (e.g., *Salmonella*) infection, rapid differentiation and proliferation of secretory ECs such as Paneth cells (which secrete anti-microbial peptides, such as defensin and lysozyme) and goblet cells [which secrete mucin and anti-microbial proteins, such as TFF3 and resistin like β (RELMβ) (also known as FIZZ1)] is accelerated to clear the pathogens (23). This countermeasure shift in epithelial stem cell differentiation is mediated by pericryptal fibroblast-produced interleukin (IL)-33 (23) (Figure 1). Differentiation into secretory ECs is ordinarily repressed by Hes1 through the Notch signaling pathway (24, 25). But in the *in vitro* assessment with intestinal organoids IL-33 acts on epithelial stem cells *via* its receptor ST2, to suppress Notch signaling and thereby activate secretory EC differentiation (23) (Figure 1). IL-1β, IL-6, tumor necrosis factor (TNF)-α and bacterial cell components (e.g., lipopolysaccharide) are involved in the stimulation of IL-33 (23), but the extent of each of their roles is still unknown and needs further investigation.

Homeostatic maintenance of epithelial stem cells is generally understood to be maintained by neighboring Paneth cell production of Wnt3, Wnt5, and EGF (26). However, in the colon where Paneth cells are lacking, mesenchymal cell production of Wnt2b works to maintain epithelial stem cells (27). In addition, mesenchymal cells are responsible for secreting Wnt-activating growth factors such as R-spondin 3 during both homeostatic and non-homeostatic conditions (28, 29). A recent study indicates that, during inflammation, CD34^+^ fibroblasts produce niche factors, including Wnt2b, Gremlin 1, and R-spondin 1, for maintenance of the intestinal stem cell niche (29) (Figure 1). The important role of mesenchymal cells in epithelial stem cell maintenance deepens their integral role in EC differentiation. These findings imply that the function of mesenchymal cells differs among location and reflects the surrounded tissues or microenvironments.

### Mucosal Repair

The intestinal mucosa is frequently threatened by environmental substances (e.g., pathogenic microorganisms, and chemicals such as alcohol) or dysbiosis of commensal microorganisms. The gut is thus equipped with multiple innate and acquired defense mechanisms (e.g., mucus, anti-microbial peptides, IgA antibodies, and Th17 cells) (30). Although these systems are essential for host protection, they concurrently cause mucosal damage, and it is therefore crucial to simultaneously initiate the mucosal tissue repairing cascade (1), which involves various factors promoting epithelial restitution followed by epithelial regeneration and differentiation (31).

Epithelial restitution occurs early on in mucosal epithelial tissue that has suffered tissue damage due to inflammatory diseases (32, 33). ECs near the damaged region lose polarity and migrate rapidly to the epithelial-deficient region, restoring the epithelial layer (32). Epithelial restitution does not appear to involve proliferation of ECs from the crypt region (1, 32); rather the process occurs through covering or sealing of the denuded area by migrating ECs (33). IL-22 has been shown to promote myofibroblast mediated epithelial repair and defense as well as epithelial stem cell protection during inflammatory bowel diseases (34, 35). Upon inflammation, helper T cells and innate lymphoid cells near the site of inflammation secrete IL-22 (36). IL-22 then activates NF-κB and AP-1 transcription factors as well as MAP kinases of myofibroblasts (34, 35). IL-22 activated myofibroblasts subsequently secreted proinflammatory cytokines (e.g., IL-6, IL-8, and IL-11) as well as MMP-1 and MMP-3 imperative to repair and remodeling (34). The IL-22 induced proinflammatory cytokines are necessary for the protection of epithelial stem cells and lack thereof has been linked to intestinal pathology and loss of epithelial barrier function (35). Additionally, chemokines (e.g., CXCL12) (37), and various other cytokines [e.g., IL-6 and transforming growth factor (TGF)- β1] (38, 39), and anti-microbial proteins (e.g., TFF3) (40) are suggested to play a role in epithelial restitution, the precise mechanism is still largely unknown. In addition, other studies have shown that during various other intestinal damages such as irradiation, Lgr5 positive cells are imperative to proper EC regeneration (41).

Alongside epithelial restitution, stimulation of fibroblasts near the inflammation site is an important process. Activation by immune cells (e.g., T cells and macrophages) and EC-produced TGF-β1 induces differentiation of fibroblasts into myofibroblasts expressing smooth muscle α-actin (αSMA) (6). Myofibroblasts specialize in the production of ECM molecules such as collagen and tenascin C, and together with fibroblasts, promote mucosal repair by appropriately adjusting the production and degradation of the ECM (42, 43). In addition, myofibroblasts produce growth factors (e.g., HGF), which induce EC proliferation, leading to migration of ECs to the repair site using ECM as a scaffold (44). Because efficient induction of myofibroblasts is essential for mucosal repair, several induction mechanisms exist other than development from activated conventional fibroblasts. For instance, differentiation from ECs (epithelial–mesenchyme transition) and endothelial cells (endothelial–mesenchyme transition) have been characterized in different tissues (e.g., kidney) (5, 45–47). Both epithelial– and endothelial–mesenchyme transitions induce migratory fibroblastic cells expressing vimentin and αSMA (5, 46). These processes are regulated by various cytokines, including TGF-β1, TNF-α, and IL-1β produced by immune cells and ECs (45).

In mucosal repair upon inflammatory bowel diseases (e.g., Crohn’s disease), FGF2 and IL-17 produced from regulatory T cells and Th17 cells, respectively, as the result of stimulatory signals caused by dysbiosis of the intestinal flora have been shown to play a critical role (48). FGF2 and IL-17 synergistically promote expression of genes involved in intestinal mucosa healing (e.g., those encoding SPRR2, IL-6, and Arg2). IL-17 also strongly influences ECs and mesenchymal cells during the tissue disruption and healing process mentioned above (48).

Transforming growth factor-β1 is an essential cytokine for wound healing and enhancement of ECM production (49). It has been recently announced to be discontinued the phase III trial; however, patients with Crohn’s disease have been treated with antisense oligonucleotides against SMAD7, which binds to the TGF-β receptor, blocking TGF-β1 signaling; inhibition of SMAD7 promotes TGF-β-induced activation of SMAD2 and SMAD3 signal transducers (50), thereby activating TGF-β1-mediated anti-inflammatory activities (50). However, chronic production of TGF-β1 continuously activates mesenchymal cells, especially fibroblasts and myofibroblasts, leading to organ fibrosis (51, 52). Fibrosis causes intestinal stricture and obstruction, and repeated intestinal resection results in short bowel syndrome (53). Although the mechanism of fibrosis induction is not fully understood and complex, excessive activation of the TGF-β1 pathway is generally considered to be a central causative element (54). Many patients with Crohn’s disease undergo surgery to relieve fibrotic complications as their disease worsens (51, 52). Temporal and spatial activation of TGF-β1 is believed to lead to the wound healing; however, sudden wound healing may progress intestinal obstruction (53). Further analysis of mesenchymal cells provides promising strategies for the control of wound healing.

## Mucosal Peripheral Education

### Mucosal Dendritic Cell Education

**Figure 2:**
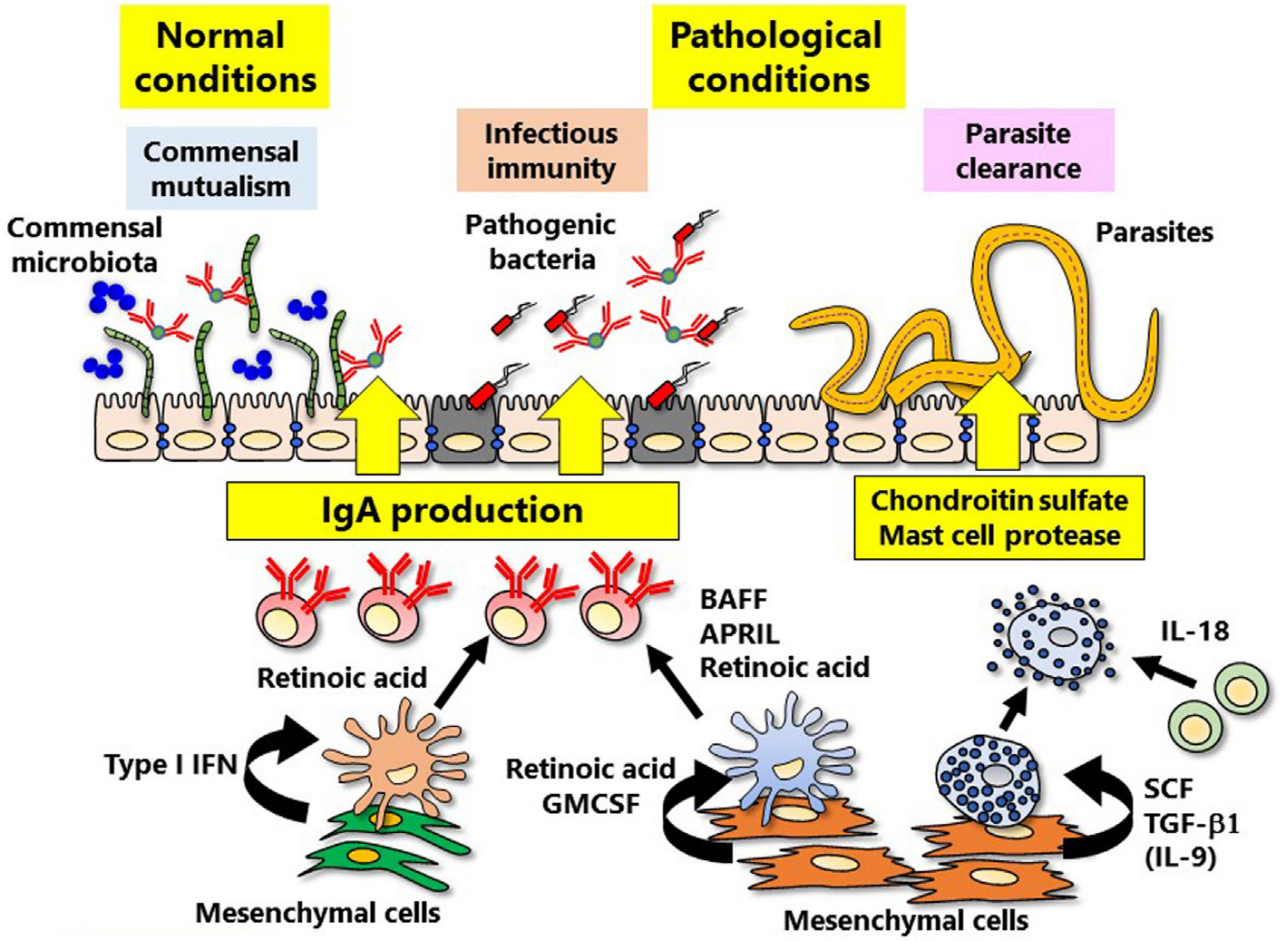
Mesenchymal cell-instructed immune cell education. Mesenchymal cells induce peripheral immune education, thereby refining intestinal-specific immune responses. IgA is involved in the both normal (commensal mutualism) and pathological (the protection against bacterial infection) conditions. Induction of IgA is directly and indirectly regulated by mucosal mesenchymal cells *via* type I IFN and retinoic acid. In addition, the defense against parasite infection mediated by mast cells is also regulated by cytokines produced from mesenchymal cells.

The intestinal tract is a special tissue that is constantly in contact with various stimuli such as microflora, foods, and metabolites. Since the intestinal tract acts as a gateway for environmental antigens and pathogenic microorganisms, the mucosal immune system must achieve the appropriate immunological balance between active and quiescent responses. The qualitative and quantitative adjustment of intestinal IgA antibody production is deeply involved in both the protection against pathogenic bacterial infection and the maintenance of the appropriate composition of commensal bacterial flora for a healthy gut environment (55). In steady state, secretary IgA antibodies are required to maintain healthy bacterial species, so called commensal mutualism (56) (Figure 2). Disruption of the mucosal immune system-mediated balancing act leads to the onset of various acute and chronic inflammatory diseases (57). In the induction of mucosal IgA antibody production, intestinal dendritic cells play a critical role by synthesizing retinoic acid (RA), which promotes antigen-specific mucosal T and B lymphocyte responses; this role is in addition to the classical role of dendritic cells in antigen presentation to T and B lymphocytes in organized inductive tissue (e.g., PPs) (58). RA-induced lymphocytes express gut-imprinting molecules such as the chemokine receptor CCR9 and the integrin α4β7, which are necessary for the preferential migration of antigen-specific lymphocytes from PPs to the lamina propria regions of intestinal tract where they produce IgA (59). RA production is peculiar to “mucosal-type” dendritic cells located in mucosa-associated lymphoid tissues (e.g., PPs), not splenic dendritic cells (58, 60). Furthermore, some mesenchymal cells can produce RA and GM-CSF (also known as CSF2), critical cytokine for generation of dendritic cells, in the vicinity of dendritic cells in the intestinal lamina propria (15) (Figure 2); from *in vitro* analysis, it has become obvious that the mesenchymal cells can convert spleen dendritic cells into “mucosal-type” dendritic cells (15). It is thus plausible to suggest the existence of a mucosal mesenchymal–dendritic cell cross-talk system that preferentially educates lymphocytes to produce IgA antibodies in the mucosa-associated tissues. Dendritic cells within the mucosal lamina propria can produce RA independently of intestinal bacteria, but RA produced from mesenchymal cells is dependent on stimulation from intestinal bacteria (15). These findings suggest that initial peripheral education machinery mediated by RA is orchestrated by the cross-communication between mesenchymal cells and commensal microbiota, which leads to the creation of a mucosal imprinting environment.

In addition to RA, cytokines that promote IgA induction such as APRIL (also known as TNFSF13) and BAFF (also known as TNFSF13B) are produced by plasmacytoid dendritic cells, another subgroup of dendritic cells within the intestinal mucosa (61) (Figure 2). Type I IFN is deeply involved in the induction of mucosal plasmacytoid dendritic cells, and it has recently been reported that intestinal mesenchymal cells are the main source of type I IFN (61). Production of type I IFN from mesenchymal cells is stimulated by intestinal bacteria (61). It is thus necessary to further verify how and what kinds of gut bacteria and/or their derived factor(s) are involved in mucosal mesenchymal cell-instructed gut-imprinting and IgA production.

### Mucosal Mast Cell Education

Mast cells undergo maturation after being distributed throughout the whole body, including gut mucosa, *via* blood from the bone marrow (13). The c-kit receptors on mast cells and the c-kit ligand (stem cell factor, SCF; also known as KITLG), are essential for maintaining mast cells; mice lacking either of these molecules have no mast cells (62). Mesenchymal cells, especially fibroblasts, are the main secretory source of SCF (13). The SCF–c-kit pathway works together with prostaglandin D2 and its receptor (DP1) pathway in the maturation of mast cells, including granule formation (63). Mast cell granules containing chondroitin sulfate and proteases (e.g., the chymase Mcpt1) are involved in the control of parasitic infections (64–66). In mice infected with an intestinal helminth, antigen–IgE complex and IL-18 activated mucosal mast cells to release chondroitin sulfate and Mcpt1 to achieve parasite expulsion. Chondroitin sulfate and Mcpt1 caused direct parasite damage and inhibited parasite invasion of ECs (67). However, inappropriate and unnecessary activation of mast cells within the mucosa, inflammation, and allergic reaction took place. For instance, proteases released from mast cells accelerate the influx of inflammatory cells (e.g., neutrophils) into the inflammatory site by weakening the tight junctions of endothelial cells (68).

Mast cells are classified into two subsets: “connective tissue type” and “mucosal type” (13). Mast cells that have heparin-containing granules are common in connective tissue, whereas those with chondroitin sulfate-containing granules are preferentially found in the intestine (13). In mast cells associated with the mucosal surface, expression of proteases Mcpt1 and -2 is particularly elevated (69). For the generation of “mucosal-type” mast cells, not only IL-9 producing T cells (so-called Th9 cells), but also gut mesenchymal cells have been shown to play a critical role (14). *In vitro* expression of heparin–Mcpt4 or chondroitin sulfate–Mcpt1 (representing “connective tissue type” or “mucosal type,” respectively) is induced by co-culturing bone marrow-derived mast cell precursors with mesenchymal cells from skin dermis or intestinal mucosa, respectively (14). Because expression of Mcpt1 is induced by TGF-β1 and IL-9 (70), only intestinal, but not skin mesenchymal cells, were able to induce Mcpt1 expression (71) (Figure 2). Taken together, these results demonstrate the presence of an intestinal mesenchymal cell-instructed “mucosal-type” mast cell development system. Further, it is interesting to hypothesize that the mesenchymal cells at different tissue locations (e.g., skin and gut) adopting the biological and anatomical characteristics of respective tissues are a major educator for the generation of “connective tissue type” and “mucosal-type” mast cells.

In summary, our new and advanced knowledge of the role of mesenchymal cell-instructed functional maturation of immunocompetent cells (e.g., dendritic cells and mast cells) will allow us to create novel strategies for the control of mucosal infection and inflammation in the near future.

## Future Perspectives

The functions of mucosal mesenchymal cells as the peripheral educator of immunological cells are critical in the development and maintenance of the intestinal homeostatic condition. Disruption of mucosal mesenchymal cell-instructed peripheral education system is likely a cause of gut pathological conditions. However, only a portion of the physiological, immunological, and pathological roles of these cells is clear, and detailed molecular and cellular mechanisms of the mucosal mesenchymal cell-instructed peripheral education system have yet to be elucidated.

Since mesenchymal cells are composed of a heterogeneous cell population, including fibroblasts, myofibroblasts, pericytes, interstitial cells of Cajal, adipocytes, and others, there remains a problem regarding the correct classification of subpopulations with specific molecular and morphological identification factors. Further investigations of the molecular role of mesenchymal cells in immune peripheral education, mucosal barrier formation, and fibrosis are required. It is thus important to elucidate the precise molecular interaction(s) between mesenchymal cells and immune cells to understand the bidirectional regulatory mechanisms. To this end, our current and future efforts aim to clarify the novel regulatory function of mesenchymal cells in the prevention of excess inflammatory reactions.

## Author Contributions

YK, DY, NS, SU, PE, TN, and HK conceived and wrote the manuscript.

## Conflict of Interest Statement

The authors are not aware of any affiliations, memberships, funding, or financial holdings that might be perceived as affecting the objectivity of this review.
